# FDA Approved Drug Library Screening Identifies Robenidine as a Repositionable Antifungal

**DOI:** 10.3389/fmicb.2020.00996

**Published:** 2020-06-03

**Authors:** Yikun Mei, Tong Jiang, Yun Zou, Yuanyuan Wang, Jia Zhou, Jinyang Li, Lin Liu, Jingcong Tan, Luqi Wei, Jingquan Li, Huanqin Dai, Yibing Peng, Lixin Zhang, Jose L. Lopez-Ribot, Rebecca S. Shapiro, Changbin Chen, Ning-Ning Liu, Hui Wang

**Affiliations:** ^1^Center for Single-Cell Omics, School of Public Health, Shanghai Jiao Tong University School of Medicine, Shanghai, China; ^2^The Center for Microbes, Development and Health, Key Laboratory of Molecular Virology and Immunology, Institut Pasteur of Shanghai, Chinese Academy of Sciences, Shanghai, China; ^3^University of Chinese Academy of Sciences, Beijing, China; ^4^Chinese Academy of Sciences Key Laboratory of Pathogenic Microbiology and Immunology, Institute of Microbiology, Chinese Academy of Sciences, Beijing, China; ^5^Department of Laboratory Medicine, Ruijin Hospital, Shanghai Jiao Tong University School of Medicine, Shanghai, China; ^6^Faculty of Medical Laboratory Science, Shanghai Jiao Tong University School of Medicine, Shanghai, China; ^7^State Key Laboratory of Bioreactor Engineering, East China University of Science and Technology, Shanghai, China; ^8^Department of Biology, The University of Texas at San Antonio, San Antonio, TX, United States; ^9^South Texas Center for Emerging Infectious Diseases, The University of Texas at San Antonio, San Antonio, TX, United States; ^10^Department of Molecular and Cellular Biology, University of Guelph, Guelph, ON, Canada

**Keywords:** *C. albicans*, robenidine, filamentation, cell wall integrity, Rlm1, antifungal agents

## Abstract

Due to the increasing prevalence of pathogenic fungal infections, the emergence of antifungal resistant clinical isolates worldwide, and the limited arsenal of available antifungals, developing new antifungal strategies is imperative. In this study, we screened a library of 1068 FDA-approved drugs to identify hits that exhibit broad-spectrum antifungal activity. Robenidine, an anticoccidial agent which has been widely used to treat coccidian infections of poultry and rabbits, was identified in this screen. Physiological concentration of robenidine (8 μM) was able to significantly inhibit yeast cell growth, filamentation and biofilm formation of *Candida albicans* – the most extensively studied human fungal pathogen. Moreover, we observed a broad-spectrum antifungal activity of this compound against fluconazole resistant clinical isolates of *C. albicans*, *as well as* a wide range of other clinically relevant fungal pathogens. Intriguingly, robenidine-treated *C. albicans* cells were hypersensitive to diverse cell wall stressors, and analysis of the cell wall structure by transmission electron microscopy (TEM) showed that the cell wall was severely damaged by robenidine, implying that this compound may target the cell wall integrity signaling pathway. Indeed, upon robenidine treatment, we found a dose dependent increase in the phosphorylation of the cell wall integrity marker Mkc1, which was decreased after prolonged exposure. Finally, we provide evidence by RNA-seq and qPCR that Rlm1, the downstream transcription factor of Mkc1, may represent a potential target of robenidine. Therefore, our data suggest that robenidine, a FDA approved anti-coccidiosis drug, displays a promising and broadly effective antifungal strategy, and represents a potentially repositionable candidate for the treatment of fungal infections.

## Introduction

Fungal infections pose a global threat to human health. Every year, billions of people around the world suffer from fungal infections, resulting in more than 1.5 million deaths. *Candida albicans* is the most frequently isolated human fungal pathogen in the clinic (Martin et al., 2003; Zaoutis et al., 2005; Pfaller and Diekema, 2007). The mortality rate of bloodstream infections caused by *C. albicans* is 40–70% (Wenzel, 1995), especially in severely immunocompromised patients. The existing arsenal of antifungals to treat these life-threatening infections is very limited, with some therapeutics exhibiting a narrow spectrum of activity, and/or severe side-effects (Pina-Vaz et al., 2004). Additionally, the emergence of antifungal-resistant fungal isolates is an increasing concern (Butler and Buss, 2006; Lam, 2007). Therefore, identifying new antifungals drugs and their targets represents an urgent need in the field.

Currently, three major classes of antifungals are used to treat fungal infections: polyenes, echinocandins, and azoles. The polyene amphotericin B binds to ergosterol in fungal cell membrane and increases the permeability of cell membrane, which results in leakage of electrolytes, amino acids, and other important substances in the cytoplasm, leading to cell death (Utz, 1964). However, the severe side-effects, especially nephrotoxicity, associated with amphotericin B limits its clinical application. The echinocandin caspofungin inhibits the synthesis of β-(1,3)-D-glucan, which results in an abnormal cell wall structure, cell wall disruption, leakage of important substances, and eventually fungal cell death. However, caspofungin is poorly absorbed orally and can only be administered intravenously at a high price, which can be accompanied by adverse reactions such as fever, local phlebitis, headache and histamine-like reactions (Neoh et al., 2018). The azole fluconazole is the most widely used antifungal drug; it reduces ergosterol synthesis in fungal cells by selectively inhibiting the activity of C14-α-demethylase, which ultimately inhibits fungal cell growth (Xu et al., 2008).

The over-use of antifungals has contributed to the emergence of drug-resistant strains of *C. albicans*. In addition to the emergence of genetically encoded drug resistance, *C. albicans* is also able to tolerate antifungal drug treatment through the formation of biofilms. Biofilms are complex communities of bacteria or fungi, aggregated on biological or abiotic surfaces, and surrounded by extracellular secretions. Biofilm formation occurs in predictable stages, including initial cellular adhesion, biofilm initiation, maturation, detachment, and diffusion. Biofilm formation can enhance a microorganisms’ ability to survive host immune attacks and tolerate treatment with antimicrobial drugs (Nobile et al., 2012). Most *C. albicans* infections are associated with biofilm formation, which leads to high morbidity and mortality rates (Nobile and Johnson, 2015; Lohse et al., 2018). *C. albicans* biofilms are comprised of cells of different cellular morphologies: yeast, hyphae, and pseudohyphae. These fungal cells are surrounded by a protective extracellular matrix, which contributes to resistance to antifungal therapy. In addition, the formation of biofilms can protect *C. albicans* from killing by the host immune system (Kuhn et al., 2002).

The fungal cell wall is critical for maintaining cell morphology, and protecting against various environmental stressors including the host immune system (Mouyna et al., 2000; Rolli et al., 2009). In *C. albicans*, the cell wall is composed of an inner layer of chitin and β-glucan (β-1,3- and β-1,6-glucan), and an outer layer of mannan and mannosyl glycoprotein (Shepherd, 1987; Kapteyn et al., 2000; Poulain, 2015). Mannan accounts for up to 40% of cell wall dry weight, and is essential for cell wall integrity, cell adhesion, and host immune recognition. Although chitin is a relatively small component of the cell wall (1–3%), it is essential for cell viability (Bates et al., 2005, 2006). Given the unique aspects of the fungal cell wall, which are not shared with human host cells, the cell wall represents a very attractive target for antifungal drug development (Cabib et al., 1982; Beauvais and Latgé, 2001; Bowman and Free, 2006).

As one of the mitogen-activated protein kinases (MAPK) in *C. albicans*, Mkc1 regulates cell wall integrity during growth and morphogenesis, and the cell wall stress response (Navarro-García et al., 1995; Diez-Orejas et al., 1997; Kumamoto, 2005). Mkc1 is activated through phosphorylation, and signaling mediated by Mkc1 can change the gene expression and accumulation of osmotic stress response factors, which is necessary for adaptation to high osmotic stress (Ene et al., 2015). Additionally, the calcineurin and high osmotic stress pathways have been shown to be involved in the fungal cell wall damage response (Delgado-Silva et al., 2014).

Due to the urgent need for the development of new antifungals, we performed a high-throughput screen of an FDA-approved compound library to identify potentially novel antifungal agents, with the consideration that repurposing currently available drugs may substantially reduce the time and effort required for antifungal development. This L4200 chemical library consists of 1,068 commercially available drugs, which target angiogenesis, and infectious, neurodegenerative, endocrine, and metabolic diseases. Since the structure and bioactivity of these chemicals are mostly known, we can expect that any drug identified during the screening will also facilitate the investigation of the antifungal mechanism of action. Through this screen, we identified robenidine: a veterinary drug used to treat coccidiosis (Molecular formula, molecular weight and molecular structure of robenidine are shown in Supplementary Figure S1). Our results show that this compound has broad-spectrum antifungal activity against *C. albicans*, as well as a diversity of clinically important fungal pathogens. Moreover, we find that robenidine exerts its activity on the fungal cell by targeting the highly conserved cell wall integrity signaling pathway. Our study suggests that robenidine may be repurposed as a promising antifungal against pathogenic fungi such as *C. albicans*.

## Materials and Methods

### Strains and Culture Conditions

The strains used in this study are shown in Supplementary Table S1. All strains were stored at −80°C. *C. albicans* cells were recovered in YPD medium (1% yeast extract, 2% peptone, and 2% glucose) and grown for 24 h at 30°C.

### Growth Curve Assay

Cells grown overnight in YPD medium were washed in PBS and diluted to an OD_600_ of 0.2 in 200 μl medium in flat-bottomed 96-well plate. The OD_600_ was obtained every 15 min in BioTek plate reader at 30°C. The standard deviation (SD) of at least three technical replicates were calculated and graphed in Graphpad Prism Software. Growth during drug exposure was assayed in YPD medium. The vehicle for Robenidine (T2549; TargetMol) was DMSO. Fluconazole (HY-B0101; MCE) was used as a positive control. All panels shown represent at least three biological replicates.

### Biofilm Formation

*Candida albicans* cells were diluted into 100 μl of RPMI-1640 medium in the 96-well plate which was sealed and incubated in the 37°C incubator for 4 h for adhesive biofilm formation. After pipetting out the supernatant, the biofilm was washed once with PBS and treated with robenidine for 24 h. To quantify biofilm formation, 100 μL XTT (A602525-0250; Sangon Biotech) solution containing 1% phenazine methosulphate (Sigma-Aldrich, United States) was added into each well and incubated at 37°C for 2–3 h. Then OD492 of the supernatant was measured using a BioTek plate reader (Li et al., 2019). For quantification of the dry weight of mature biofilm, *C. albicans* cells were diluted to 1 ml RPMI 1640 medium and sealed in 12-well plate. The tailored silica film was placed at the bottom of the 12-well plate, labeled and weighed beforehand, and incubated in an incubator at 37°C for 24 h. After robenidine treatment for 24 h, the silica film of each well was dried in an oven for 5 h at 60°C, with the biofilm attached on the top. After weighing, the weight of the silica film was subtracted to obtain the weight of biofilm.

### Filamentation Assay

Cells grown overnight in YPD were washed twice in PBS and diluted to an OD_600_ of 0.001 in YPD medium with 10% bovine serum, M199 medium, and Spider medium, respectively. After robenidine treatment at 37°C (a strong filament inducing condition) (Liang et al., 2016; Romo et al., 2018) for 2 and 4 h, cells were photographed under the NIKON microscope.

### Cell Wall Stress Response

Cells incubated overnight at 30°C were washed twice with PBS and diluted to OD_600_ of 0.2 with 8 (μM robenidine treatment for 4 hours. Then the cells with OD600 = 0.5 were spotted onto YPD plates containing CaCl_2_ (C3306-250G; Sigma/flu/Ald), Caffeine (N2379-20mg; APExBIO), Congo Red (HY-D0236-500mg; MCE), Calcofluor White (18909-100ML-F; Sigma-Aldrich), or DMSO at the indicated concentrations. Photos were taken after 24 h incubation at 30 or 37°C.

### Alcian Blue Binding Assay

An Alcian Blue binding assay was used for the detection of cell wall integrity (Hobson et al., 2004). Overnight cell cultures were diluted to OD_600_ = 0.2 and grown for 4 h at 30 or 37°C. Then robenidine was added to a final concentration of 8 and 16 μM. After 4 h, cells were stained with 30 μg/ml Alcian Blue (A3157-10G; Sigma/flu/Ald) for 10 min. Then the OD_620_ of the supernatant was measured and the cell pellets were photographed. The amount of Alcian Blue binding was calculated by subtraction of unbound dye in the supernatant. The data was analyzed by a one-way ANOVA.

### Measurement of Cell Wall Components

Overnight cell cultures were diluted to OD_600_ = 0.2 and robenidine was added to a final concentration of 8 and 16 (μM, and grown for 4 hours. Fungal cells collected were then fixed with 4% PFA for 1 hour and washed with PBS. For chitin test: after staining with 30 (μg/ml CFW for 1 hour, 10 (μl cell suspensions were pipetted onto the slide and photographed under the NIKON microscope. For mannan test: after staining with 50 (μg/ml ConA-488 for 1 hour incubate at 37(C, cells were washed and filtered into a single cell with silica gel. The fluorescence intensity was analyzed through flow cytometer (BD LSRFortessa).

### Western Blot

The cell lysis, protein extraction and western blot procedures were performed as described in Liu et al. (2017). The antibodies used are listed in Supplementary Table S2. For densitometry, Image J software^1^ was used as in Liu et al. (2017).

### Transmission Electron Microscopy (TEM)

Overnight saturated cell cultures were diluted in YPD medium to get an OD_600_ of 0.2. Robenidine was added to washed cells for 4 h and fixed with 2.5% glutaraldehyde in PBS for 2.5 h. After three times’ immobilization with 1% osmium acid, the cells were treated sequentially with ethanol, acetone, embedding solution and kept in 37, 45, and 60°C for 48 h. The ultrathin slides were stained with 3% uranium acetate and lead citrate and observed under electron transmission microscope (JEM-1230).

### LDH-Based Cytotoxicity Assay

FaDu (human pharyngeal squamous cell carcinoma) cells were incubated in MEM medium (10% FBS) for 24 h at 37°C and washed three times with PBS. Then the cells were seeded in a 96 well-plate with 100 (μL MEM for another 24 h. After addition of robenidine, cell cytotoxicity was measured using the lactate dehydrogenase (LDH) Cytotoxicity Detection Kit (WST and Ck12-2000T). Cell viability was determined by adding 3-(4,5-dimethylthiazol-2-yl)-2,5′-diphenyltetrazolium bromide solution (MTT; Sigma-Aldrich; final concentration 0.3 mg/ml) into cell culture plates and incubated in the dark for 2 h. The medium was then removed and added with formazan, and the concentration was determined by absorbance at 492 nm. The survival percentage was calculated accordingly (the mean survival rate of cells incubated with culture medium alone was set as 100%).

### RNA-Seq

Cells collected from overnight cultures were diluted to OD_600_ of 0.2 and treated with 8 μM robenidine for 1.5 h. RNA was then extracted and loaded for Illumina PE 2 × 150 double terminal sequencing established in Ploya library. Cutadapt and fastp software were adapted to clean the low-quality raw data of Polya and adapter sequence. Then the sequence was compared with NCBI reference database of C. albicans by bowtie2 software. Finally, the RNA assembly was used to get the expression of each transcript. The mean TPM value of each gene was normalized for differential expression analysis and the statistical significance was calculated by *t*-test. The gene set enrichment analysis was performed with the KEGG database of C. albicans including genes with *P*-value < 0.05 and *Q*-value < 0.2.

### RT-qPCR

Cells grown overnight were diluted into YPD with 8 μM robenidine and harvested after 1.5 h. RNA was extracted with chloroform and isopropanol. RT-qPCR procedures were performed as previously described (Liu et al., 2017).

### Statistical Analysis

All experiments were done for at least three biological replicates with three technical replicates. Data were expressed as mean ± SD unless otherwise specified. The survival curve was statistically analyzed by the Kaplan–Meier method (log-rank test, GraphPad Prism). For the other comparison, unpaired Student’s *t*-test was used.

## Results

### Robenidine Significantly Inhibits the Yeast Growth of *Candida albicans*

**FIGURE 1:**
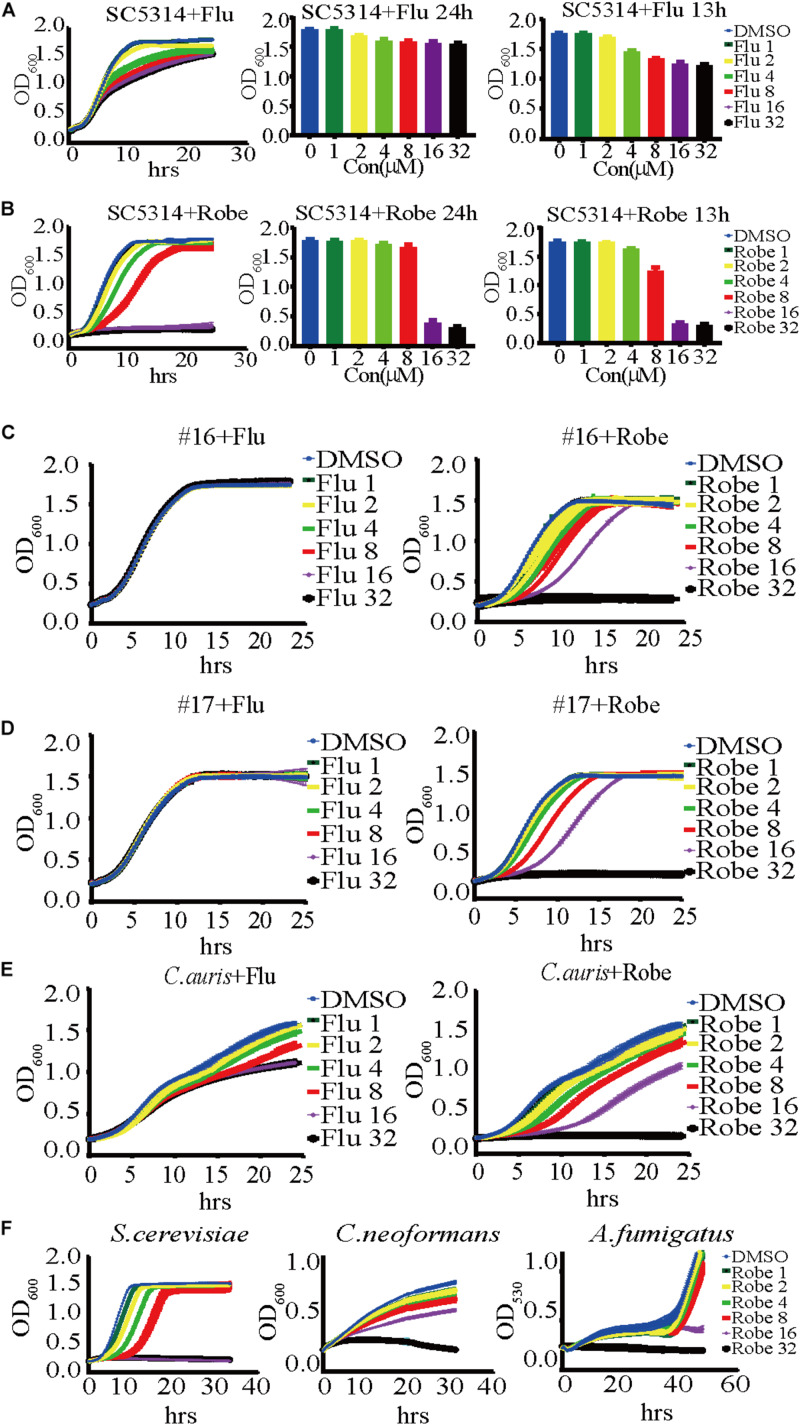
A dose-dependent inhibitory effect of robenidine and fluconazole on the growth of *Candida albicans*, fluconazole-resistant *C. albicans*, the emerging fungal pathogen *C. auris* and other fungal species. **(A)** (Left) A time-course growth of *C. albicans* in the presence of fluconazole at different concentrations. The OD_600_ was obtained every 15 min in BioTek plate reader last for 24 h. (middle and right) Effects of fluconazole at different concentrations on the growth of *C. albicans* after 13-h incubation or 24-h incubation. **(B)** (Left) A time-course growth of *C. albicans* in the presence of robenidine at different concentrations. The OD_600_ was obtained every 15 min in BioTek plate reader last for 24 h. (middle and right) Effects of robenidine at different concentrations on the growth of *C. albicans* after 13-h incubation or 24-h incubation. “Flu” stands for fluconazole and “Robe” stands for robenidine. **(C)** Growth curves of the fluconazole resistant isolate #16 after treatment with fluconazole or robenidine. **(D)** Growth curves of fluconazole resistant isolate #17 after treatment with fluconazole or robenidine. **(E)** Growth curves of *C. auris* after treatment with fluconazole or robenidine. **(F)** Dose-dependent growth inhibition of robenidine observed in testing its antifungal activity against *Saccharomyces cerevisiae*, *Cryptococcus neoformans*, or *Aspergillus fumigatus*. Each growth curve experiment has been repeated more than 4 times.

We screened 1,068 compounds in the L4200 chemical library (TargetMol) for their antifungal activity against *C. albicans*, using a standard growth curve assay that measures optical density over 24 h. SC5314 is the standard lab reference strain of *C. albicans*, which was used in this study (Romo et al., 2019). Fluconazole, a clinically important and commercially available antifungal drug, was used as a positive control for fungal growth inhibition. The candidate compounds showing equal or stronger growth inhibitory potency against SC5314, when compared to fluconazole, were selected for further study. From our screening, the drug identified with the largest inhibitory effect against *C. albicans* was robenidine. As shown in Figure 1A, both fluconazole and robenidine inhibit the growth of SC5314 in a dose dependent manner. Strikingly, robenidine suppresses the growth of *C. albicans* more effectively than fluconazole at the same concentration (16 μM) (Figure 1B). And this concentration (16 μM), as well as at higher concentration tested (32 μM), robenidine was not toxic to either endothelial cells or macrophage cells (Supplementary Figure S2). At 32 μM concentration (11.86 μg/ml), robenidine, but not fluconazole, was able to completely inhibit growth of *C. albicans*. These results indicate that robenidine is effective in inhibiting *C. albicans* growth.

### Robenidine Exhibits a Broad-Spectrum Antifungal Activity

The emergence of antifungal resistance amongst fungal pathogens severely endangers human health. To test whether robenidine possesses broad spectrum antifungal activity against drug-resistant *Candida* strains, we investigated the inhibitory effects of robenidine on two clinical isolates of fluconazole-resistant *C. albicans*, referred to as isolates #16 and #17 (Tong et al., 2016). As shown in Figures 1C,D, fluconazole alone cannot inhibit the growth of these two drug-resistant isolates, even at the highest concentration that was tested in the study (32 μM). In contrast, robenidine drastically inhibits the growth of the two fluconazole resistant isolates in a dose-dependent manner, even at 8 μM of the drug. Next, we extended the examination of antifungal properties of robenidine to the emerging fungal pathogen *Candida auris*, which has received significant attention as a multidrug resistant pathogen causing major outbreaks in health care facilities across the world, leading to high mortality rates (Wang et al., 2018). Here, we used the Chinese clinical isolate of *C. auris* first reported in 2018 (Wang et al., 2018), and tested if *C. auris* growth could be inhibited by robenidine. Surprisingly, we found that robenidine was much more efficient than fluconazole in its ability to inhibit the growth of *C. auris* (Figure 1E). Interestingly, the inhibitory efficacy of robenidine was dose-dependent, as we observed a complete abolishment of growth when the concentration was increased to 32 μM. Therefore, robenidine is highly effective at inhibiting the growth of fluconazole-resistant *C. albicans* clinical isolates, as well as the emerging drug-resistant fungal pathogen *C. auris.*

Finally, we tested the inhibitory activity of robenidine against the human fungal pathogens *Cryptococcus neoformans* (strain H99) and *Aspergillus fumigatus* (strain AF293), as well as the model yeast organism *Saccharomyces cerevisiae* (strain BY4742). We found that they were all effectively inhibited by robenidine, especially at the highest concentration of 32 μM (Figure 1F). Furthermore, the inhibitory concentration of robenidine for all tested species was extremely low, ranging from 1 to 2 μM (Figure 1F). Overall, our results reveal that robenidine exhibits an extensively broad antifungal spectrum toward a variety of human fungal pathogens, suggesting its potential use as an effective antifungal agent.

### Robenidine Inhibits Filamentation and Biofilm Formation of *C. albicans*

**FIGURE 2:**
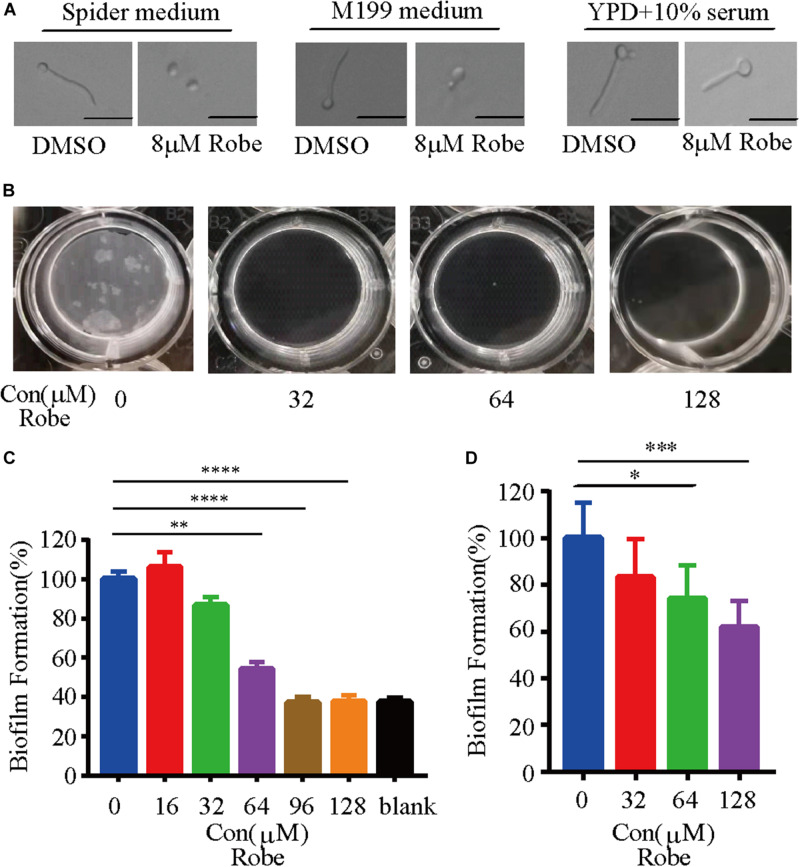
Inhibition of *C. albicans* filamentation induction under different hyphal-inducing conditions and biofilm formation by robenidine. **(A)** Cells were inoculated into different hyphal-inducing medium, such as Spider, M199, and YPD supplemented with 10% serum in the presence or absence of robenidine and grown for 2 h at 37°C. Scale bar, 20 μm. Results shown are representative result of 100 cells. **(B)** Early stage biofilm formation of *C. albicans* in RPMI medium supplemented with different concentrations of robenidine in a 12-well plate and photos were taken after discarding supernatant. **(C)** *In vitro* activity of different concentrations of robenidine on the early stage biofilm formation after incubation in 96-well plate at 37°C for 4 h, as determined by XTT colorimetric readings at OD_492_. Error bars represent SDs of 4 technical replicates. Statistical significance was determined using the paired Student’s *t*-test. **p < 0.01, ****p < 0.0001. **(D)** Mature biofilm formation of *C. albicans* in RPMI medium supplemented with different concentration of robenidine in a 12-well plate. The dry weights of the biofilms were then measured. Error bars represent SDs of 6 technical replicates. Statistical significance was determined using the paired Student’s *t*-test. *p < 0.05, ***p < 0.001. Each filamentation and biofilm experiment has been repeated more than 5 times.

The ability to transition between hyphal and yeast cellular morphologies is critical for *C. albicans* pathogenesis (Sudbery et al., 2004; Berman, 2006; Sudbery, 2011; Gow et al., 2012). Therefore, we tested the effect of robenidine on *C. albicans* filamentation in three different hyphal induction media. As expected, *C. albicans* cells developed hyphae after 2 h incubation in hyphae-inducing media, including Spider media, M199, and YPD supplemented with 10% serum (Sudbery et al., 2004). However, hyphal growth was significantly inhibited by addition of robenidine to the indicated medium (Figure 2A), with the exception of serum media, where robenidine exhibited only a modest inhibitory effect on filamentation. Therefore, these results suggest that robenidine is able to efficiently inhibit the filamentous growth of *C. albicans*, which is a key virulence trait.

Like many microbes, biofilm formation by pathogenic fungi represents the most common type of growth in nature, and is critical for the development of clinical infections. *C. albicans* biofilms are highly tolerant to treatment with most antifungal agents (Finkel and Mitchell, 2011). Given that filamentation is an important feature of biofilm development (Nobile and Johnson, 2015), and treatment with robenidine can efficiently repress hyphal growth (Figure 2A), we hypothesized that this compound might have an inhibitory effect on biofilm formation. Indeed, we found that robenidine significantly inhibits biofilm formation in *C. albicans* when biofilms were established at 37°C for 24 h in RPMI media (Figure 2). As shown in Figure 2B, we can visually monitor the disappearance of biofilms in the presence of different concentrations of robenidine. Inhibitory activity of robenidine on biofilms of *C. albicans* was further examined and measured using the XTT colorimetric assay, which monitors biofilm metabolic activity (Jin et al., 2003; Li et al., 2019). Interestingly, treatment with 64 μM robenidine leads to a dramatic reduction of biofilm adhesion, up to 50% (Figure 2C). In addition, by comparing the dry weight of biofilms before and after treatment with different concentrations of robenidine, we found that robenidine displayed inhibitory activity against mature biofilms (Figure 2D). Biofilm inhibition by robenidine even at above MICs is minor and it is mostly due to its antifungal activity. No growth means no biofilm formation. Thus this is about a characteristic of biofilm, but not biofilm inhibition by Robenidine. Taken together, this suggests that robenidine inhibits both filamentation and biofilm formation in *C. albicans*.

### Cell Wall Integrity Is Damaged by Robenidine

**FIGURE 3:**
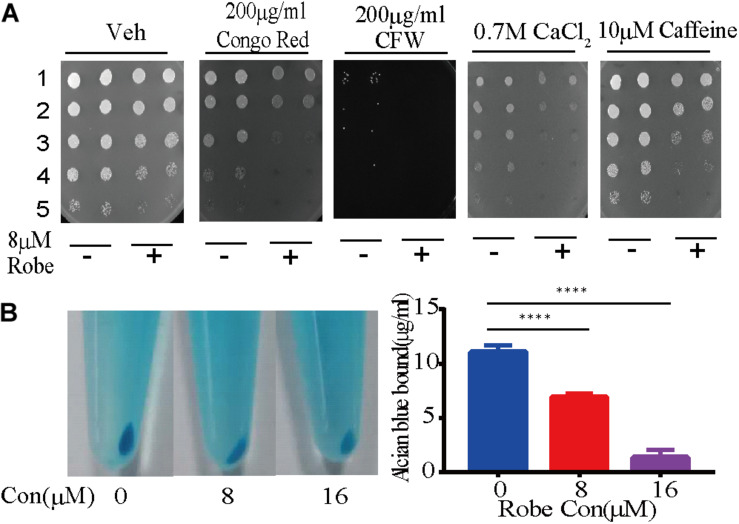
Robenidine disrupts cell wall integrity of *C. albicans*. **(A)** Cell growth under various conditions of cell wall stress. Cells treated with 8 μM robenidine were plated onto the YPD plates with different chemicals. “1” stands for OD_600_ of 0.5, “2” stands for OD_600_ of 0.1, “3” stands for OD_600_ of 0.02, “4” stands for OD_600_ of 0.004, “5” stands for OD_600_ of 0.0008. **(B)** Alcian Blue staining of the cells treated with robenidine. After robenidine treatment, cells were stained with Alcian Blue and photos were taken after centrifugation (left). The OD_620_ of supernatant was then measured by a plate reader. Left panel: Photos of cells stained with Alcian Blue; Right panel: percentage of the Alcian Blue dye binding to the cells. Error bars represent SDs of 3 technical replicates. ****p < 0.0001. Both cell wall integrity tests have been performed 4 times.

As a commensal organism associated with human hosts, *C. albicans* is frequently confronted with stressors from host microenvironments. Thus, this fungus has developed various stress responsive signaling pathways to withstand these stressors. To investigate how robenidine impacts the sensitivity to different stressors, we spotted robenidine-treated cells in 1:5 serial dilutions onto YPD plates containing different stressors (Figure 3A). The stress conditions tested include: 0.7 M calcium chloride, 200 μg/ml Calcofluor White (CFW), 200 μg/ml Congo Red, and 10 μM caffeine. We found that robenidine treated cells were hypersensitive to cell wall stressors including Congo Red and Calcofluor White. This assay was performed at both 30 and 37°C (Supplementary Figure S3A). This indicates that robenidine may interfere with the cell wall structure of *C. albicans*, as fungal growth is inhibited in response to cell wall stressors.

Next, we hypothesized that the antifungal mechanism conferred by robenidine might be related to the cell wall integrity signaling pathway. As the major component of the *C. albicans* cell wall, the negatively charged phosphomannan can be quantified by monitoring binding of the cationic phthalocyanine dye Alcian Blue. This has now been developed as a widely used quantitative assay to assess cell wall integrity. To test this hypothesis, we first evaluated the effect of robenidine on fungal cell wall integrity by staining the *C. albicans* cells with Alcian Blue (Hobson et al., 2004; Li et al., 2009; Zhang et al., 2016; Liu et al., 2020). Using this assay, we observed a dose-dependent reduction of the binding of Alcian Blue to the cell wall after robenidine treatment, as shown by the light blue color of the cell pellets (Figure 3B). This assay was performed at both 30 and 37°C (Supplementary Figure S3B). Therefore, we suggest that robenidine disrupts the integrity of the cell wall structure in *C. albicans*.

**FIGURE 4:**
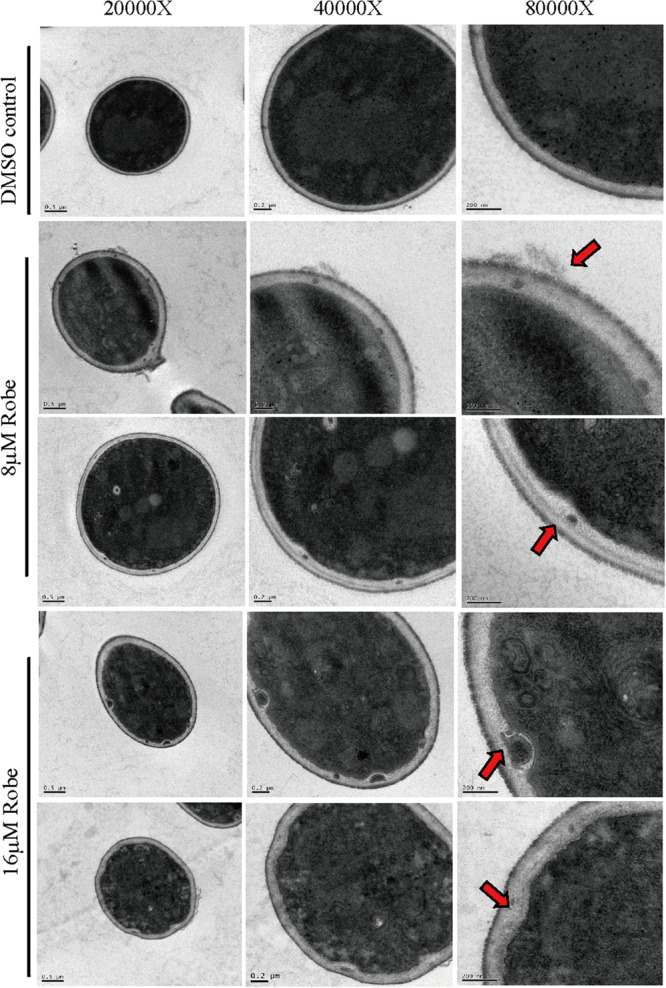
The cell wall structure was detected by TEM after treatment by robenidine. Different scale bars (0.5 μm, 0.2 μm, and 200 nm) corresponding to different magnification of TEM (20,000×, 40,000×, and 80,000×).

To monitor the physical structure of the cell wall, *C. albicans* cells treated with robenidine were further investigated by transmission electron microscopy (TEM). In the absence of robenidine, the cell wall was intact, and the cell outline arc was smooth and uniform. However, upon treatment with a low dose of robenidine (8 μM), the cell wall became thinner and the cytoplasm leaked into the space between the plasma membrane and cell wall (Figure 4). The impairment of the cell wall structure was further enhanced upon treatment with a higher dosage of robenidine (16 μM), where we observed a significantly atrophied cell wall, with additional cytoplasmic fluid leakage (Figure 4).

**FIGURE 5:**
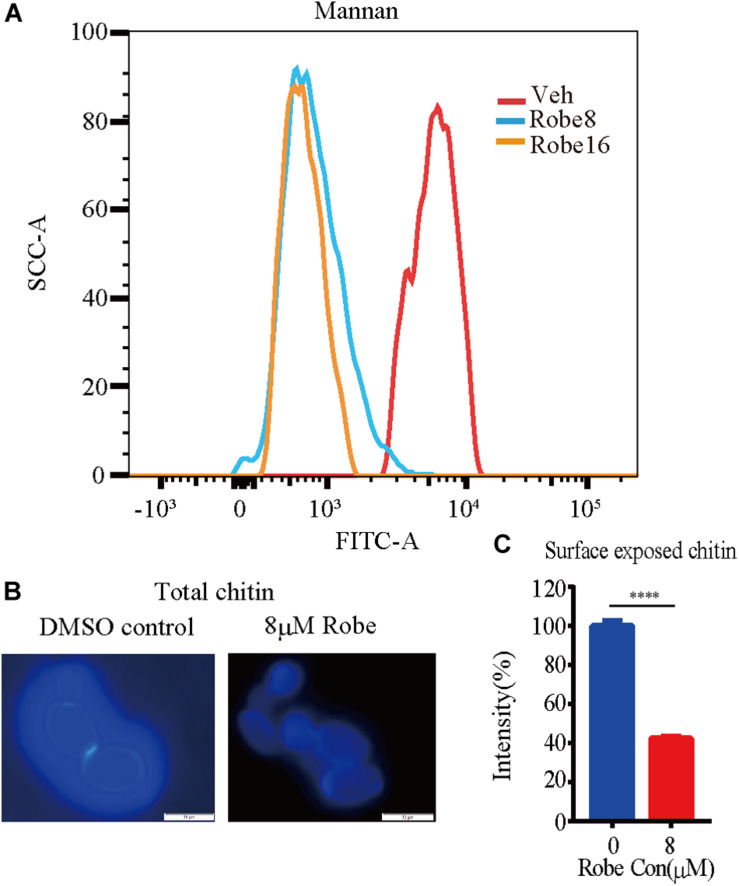
Increased mannan and decreased chitin content after treatment with robenidine. Cells were incubated in YPD supplemented with or without robenidine (8 μM) for 4 h. Then mannan and chitin content of cell wall were measured accordingly. **(A)** Calcofluor White staining for cell wall chitin. **(B)** Surface exposed chitin stained by WGA-lectin fluorescent antibody. ****p < 0.0001. **(C)** Flow cytometry analysis of cell wall mannan stained by ConA-488. The detection of mannan and chitin has been repeated three times.

The cell wall of *C. albicans* is mainly composed of β-1,3- and β-1,6-glucan, chitin and mannan (Kapteyn et al., 2000; Mouyna et al., 2000; Rolli et al., 2009; Poulain, 2015). To investigate the specific cell wall components altered by robenidine, we stained the total chitin with Calcofluor White (CFW) and the total mannan with ConA-488. In addition, the chitin exposed in the surface was stained by WGA-lectin. As shown in Figure 5A, the mannan content in the cell wall was increased after treatment with robenidine, as measured using FACS analysis. However, the amount of chitin components is reduced by robenidine, as the fluorescent staining at the junction of mother/daughter cells was diminished after treatment with robenidine (Figure 5B). This phenotype was further supported by a quantitative analysis for detection of chitin components exposed on the cell surface (Figure 5C). Thus, our results suggest that the cell wall composition, including both mannan and chitin, are altered by robenidine.

### Robenidine Targets the Cell Wall Integrity Signaling Pathway via Mkc1 Phosphorylation

**FIGURE 6:**
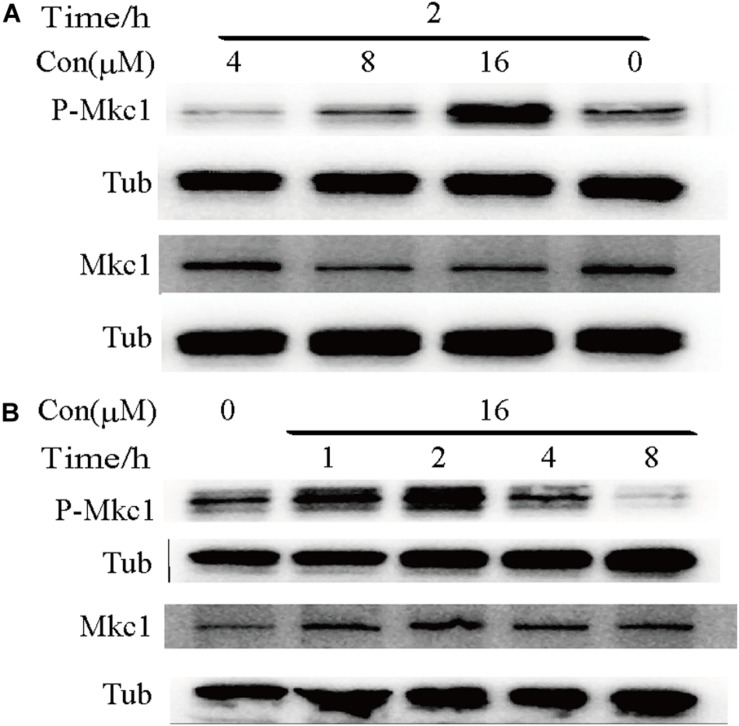
Phosphorylation of Mkc1 after robenidine treatment. **(A)** Western blot of WT (SC5314) cells treated with robenidine (0, 4, 8, and 16 μM) grown in YPD for 2 h and probed for P-Mkc1, Mkc1, and tubulin. Tubulin was used as a loading control. **(B)** Western blot of WT (SC5314) cells with robenidine (16 μM) grown in YPD for 1–8 h and probed for P-Mkc1, Mkc1, and tubulin. Tubulin was used as a loading control. All the Western blot have been repeated 3 times.

To determine whether robenidine affects the cell wall structure by targeting cell wall integrity signaling, we monitored Mkc1 phosphorylation after robenidine treatment. *C. albicans* cells were treated with different concentrations of robenidine and levels of phosphorylated Mkc1 were determined by western blot analysis. As shown in Figure 6, we found a dose-dependent induction of Mkc1 phosphorylation following the treatment of robenidine, with a peak induction at 16 μM (Figure 6A). Mkc1 phosphorylation was reduced following long-term exposure to robenidine (after treatment for 8 h) (Figure 6B). Thus, robenidine initially activates Mkc1 phosphorylation, and this is gradually decreased after prolonged treatment.

### Robenidine Regulates *RLM1* Expression Downstream of Mkc1

**FIGURE 7:**
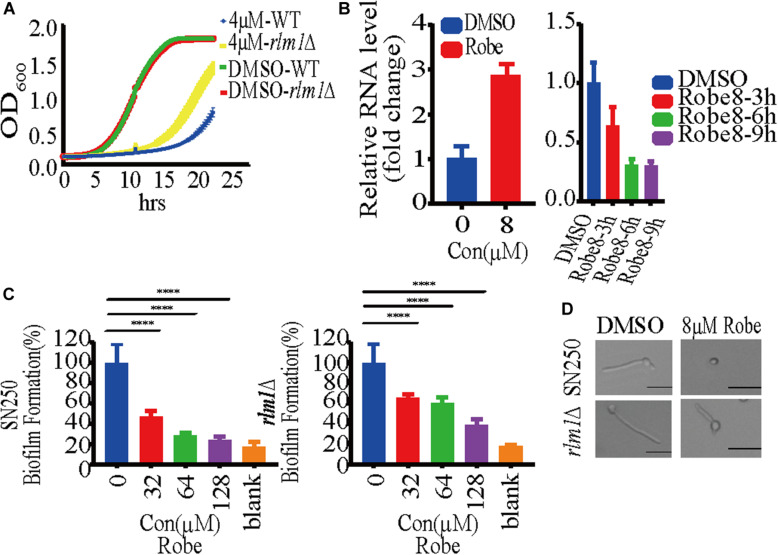
Robenidine regulates cell wall integrity pathway through Rlm1. **(A)** WT (SN250) and *rlm1* null mutant were exposed to 4 μM robenidine and OD_600_ was monitored every 15 min. **(B)** Quantitative RT-PCR analysis of *RLM1* expression after treatment of 8 μM robenidine for 90 min, 3 h, 6 h, and 9 h. Error bars indicate standard deviation based on three technical replicates. **(C)** Biofilm formation of WT (SN250) and *rlm1* null mutant in RPMI medium supplemented with different concentrations of robenidine. Cells were incubated at 37°C for 24 h and stained with XTT solution. Photos were taken after incubation for 2.5 h. **(D)** Effect of robenidine on filamentous induction of WT (SN250) and *rlm1* null mutant under M199 medium. Scale bar, 20 μm. Results shown are representative result of 100 cells. Each of these experiments had 3 repetitions of data.

Given that Mkc1 modulates several effectors in the cell wall integrity pathway, we next explored the target of robenidine by RNA-seq. Cells treated with robenidine at different time points were collected and RNA was extracted. RNA-seq analysis revealed that the expression of the transcription factor Rlm1 (Supplementary Figure S4), a downstream effector of Mkc1, was increased up to eightfold after treatment with robenidine for 90 min. Rlm1 plays an important role in maintenance of cell wall integrity (Oliveira-Pacheco et al., 2018). Interestingly, the expression of *RLM1* decreased when *C. albicans* was exposed to robenidine over a prolonged period of time (3–9 h). The transcript level of *RLM1* was further verified by RT-qPCR (Figure 7B), and assays were performed at both 30 and 37°C (Supplementary Figure S3C).

**FIGURE 8:**
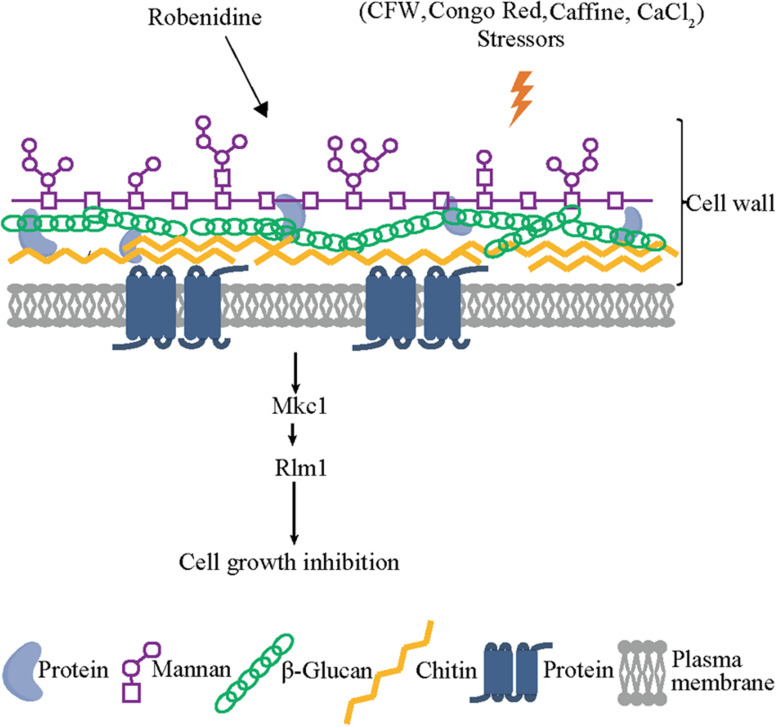
Robenidine disrupts both mannan and chitin of the cell wall components and targets Rlm1 downstream of Mkc1 which is phosphorylated to regulate cell wall integrity.

Major components of the fungal cell wall, including mannan, are significantly decreased upon depletion of *RLM1* (Delgado-Silva et al., 2014), which is consistent with our observation of altered mannan content in robenidine treating cells (Figure 5). Therefore, we speculate that Rlm1 may be one of the major gene targets of robenidine. To test this possibility, we compared the growth of both WT and *rlm1* null mutant after robenidine treatment. We found that the growth defect caused by robenidine treatment could be partially rescued upon deletion of *RLM1* (Figure 7A). In addition, WT and *rlm1* mutant strains displayed similar levels of filamentation. However, when treated with robenidine, the *rlm1* mutant retained the ability to filament while the WT strain was unable to form hyphae (Figure 7D). Additionally, the *rlm1* null mutant was able to form more robust biofilms in the presence of robenidine compared to the WT strain (Figure 7C). Altogether, our results suggest Rlm1 as the potential target of robenidine in the maintenance of cell wall integrity (Figure 8).

Thus, robenidine exerts a cell wall remodeling effect on *C. albicans* by disrupting both chitin and mannan compositions via *RLM1* expression, mediated by the Mkc1 phosphorylation pathway. This provides a possible mechanism by which robenidine disrupts cell wall integrity and exerts its antifungal activity (Figure 8).

## Discussion

Fungal infections by *C. albicans* pose a major threat to an increasing number of at-risk patients. The high morbidity and mortality rates associated with these infections points to the lack of treatment options. In particular, the scarcity of our current antifungal arsenal calls for the development of novel antifungal drugs. Repurposing FDA approved drugs as antifungal agents represents an attractive strategy.

As a veterinary antiparasitic against coccidiosis, robenidine has been widely used in animals including chicken and rabbits (Kantor et al., 1970; Dorne et al., 2013). But the anticoccidial mechanism of robenidine has remained elusive. Furthermore, the antifungal activity of robenidine has never been previously explored. In this study, our findings revealed that robenidine can be repurposed as an antifungal drug and may serve as a powerful strategy to inhibit diverse fungal pathogens, including antifungal-resistant isolates.

As part of this study, we observed that 10% serum appeared to delay the filamentation inhibition by robenidine. Since serum is mostly found to induce filamentation in *C. albicans* (Enjalbert and Whiteway, 2005; Romo et al., 2017; Su et al., 2018), we speculated this serum-induced filamentation could temporarily weaken the inhibitory effect by robenidine on certain filamentation-regulating genes (Bar-Yosef et al., 2018; Peroumal et al., 2019). However, the exact mechanism for this serum interference of the antifungal effect of robenidine remains unknown and will require further investigation. Also, we cannot rule out the possibility of serum interacting with robenidine directly. Thus, the possibility of robenidine interacting with serum and/or other filament-inducing agents needs to be further analyzed.

The fungal cell wall represents an attractive target for the development of antifungal drugs (Mouyna et al., 2000; Bates et al., 2006). Sensitivity to Congo red, caffeine, Calcofluor White and CaCl_2_ are widely used for the identification of cell wall defects. For example, CFW and Ca^2+^ are activators of the compensatory signaling pathway of chitin synthesis (Munro et al., 2007). Our observations are consistent with previous results from Bates et al. (2005). We demonstrate that robenidine significantly disrupts cell wall components chitin and alters the amount of mannan. This cell wall remodeling reflects the disruption of cell wall components. Gene expression analysis performed in cells growing under robenidine treatment identified the transcription factor Rlm1, which was significantly upregulated upon robenidine treatment. Rlm1 is reported to be involved in cell wall biogenesis for maintenance of cell wall integrity (Alam et al., 2011; Delgado-Silva et al., 2014; Oliveira-Pacheco et al., 2018). The increase of mannan component and decrease of chitin content were also consistent with the role of Rlm1 on regulation of cell wall integrity (Delgado-Silva et al., 2014). This provides a foundation for further mechanistic study of Rlm1 in robenidine mediated cell wall disruption.

The significant inhibitory effect of robenidine on fungal cells suggests that future work should test its potential therapeutic effect in animal models of disseminated fungal infection. Other factors could further be optimized to increase the efficacy of robenidine in a clinical setting, including structure optimization, and different drug delivery systems to ensure optimal drug solubility and affinity.

## Data Availability Statement

The datasets generated for this study can be found in the NCBI Biosample, https://www.ncbi.nlm.nih.gov/biosample, accession number: PRJNA598024.

## Author Contributions

CC, N-NL, and HW conceived and designed the study. JL-R, CC, N-NL, and HW performed data analysis and wrote the manuscript. YM, TJ, YZ, YW, JZ, JinyL, JT, LW, JingL, and HD conducted all the experiments and performed the statistical analysis of the data. YM, YP, HW, LZ, JL-R, RS, CC, N-NL, and HW discussed the experiments and results.

## Conflict of Interest

The authors declare that the research was conducted in the absence of any commercial or financial relationships that could be construed as a potential conflict of interest.

## Footnotes

1 https://imagej.net/Downloads
