# Supplementary material for: FDA Approved Drug Library Screening Identifies Robenidine as a Repositionable Antifungal
Source: Front Microbiol. 2020 Jun 3;11:996. doi: 10.3389/fmicb.2020.00996 (PMC7283467; doi:10.3389/fmicb.2020.00996)
Supplement: Supplementary file 1 [file Table_1.pdf]

Supplementary data

Table S1. List of strains used in this study.

Strain name	Discription	Reference
SC5314	WT strain of *Candida albicans* from Dr. Julia R. Koehler	1
Fluconazole resistant strain #16	Fluconazole-resistant clinical isolate of *Candida albicans* #16	2
Fluconazole resistant strain #17	Fluconazole-resistant clinical isolate of *Candida albicans* #17	2
Candida auris	*Candida auris* strain from Dr. Hui Wang	3
AF293	*Aspergillus fumigatus* strain from Dr. Ling Lu	4
BY4742	*Saccharomyces cerevisiae* strain from Dr. Jinqiu Zhou	5
H99	*Cryptococcus neoformans* strain	6
SN250	WT of *Candida albicans,* used to knock out *RLM1*	7

Table S2. List of primers and antibodies used in this study.

Primer name	Purpose	Sequence 5’ to 3’
Rlm1F1	Confirmation of Rlm1 deletion	GCACCTCAAGATTGGCCATC
Rlm1R1	Confirmation of Rlm1 deletion	ACCATCACCAGCATCACTCA
Rlm1F2	Forward for RT-PCR of Rlm1	TGGTTTACCGACAGGGACAC
Rlm1R2	Reverse for RT-PCR of Rlm1	GATGGCCAATCTTGAGGTGC

Antibodies Purpose	Antigen recognized	Species	Source or Reference
Loading control	Tubulin	Rat	Abcam, cat. # ab6161
Secondary	Rabbit Ig	Rabbit	Santa Cruz Biotechnology, cat. # 2370
Secondary	Rat Ig	goat	Santa Cruz Biotechnology, cat. # 97057
Primary	P-Mkc1	Rabbit	Cell signaling Technology cat. # 4370L
Primary	Mkc1	Rabbit	Cell signaling Technology cat. # 4695S

Molecular formula： $C_{15}H_{14}Cl_3N_5$

Molecular weight： 370.66 g/mol

Molecular structure：

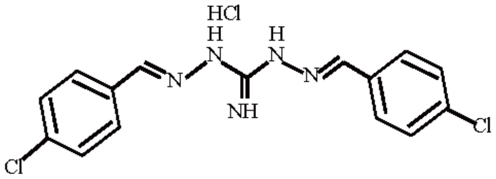

Figure S1. Molecular formula, molecular weight and molecular structure of robenidine.

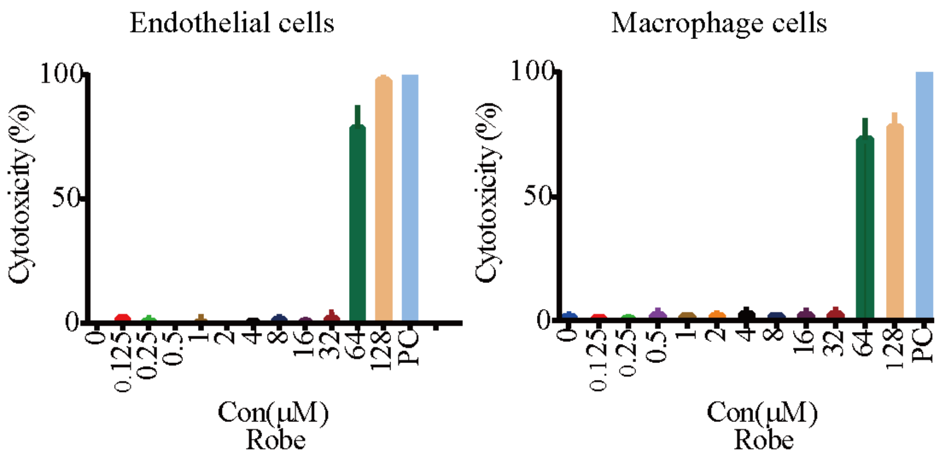

Figure S2. Cytotoxicity of robenidine to FaDu endothelial cells and RAW-BLUE macrophage cells. Cells incubated with robenidine for 24 hours were measured with LDH assay. PC represents positive control group. Each LDH assay has been repeated 3 times.

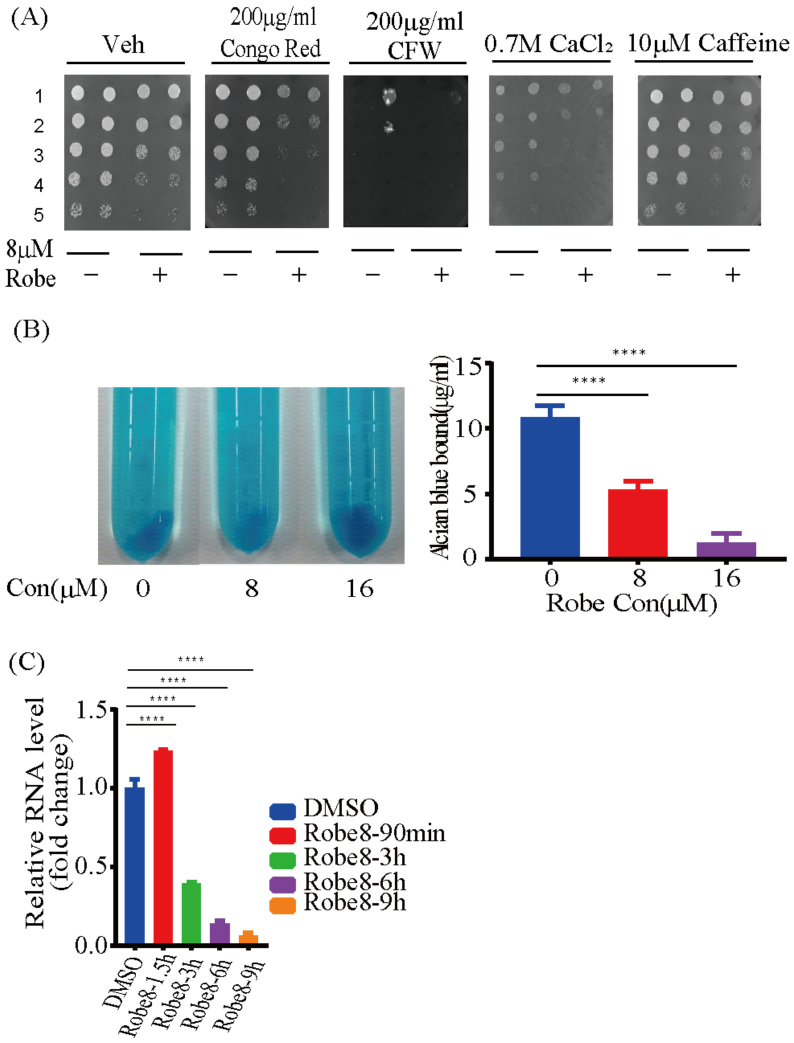

Figure S3. Robenidine disrupts cell wall integrity of *C. albicans* at 37 ℃. A) Cell growth under various conditions of cell wall stress. Cells treated with 8 µM robenidine were plated onto the YPD plates with different chemicals. "1" stands for OD_{600} of 0.5, "2" stands for OD_{600} of 0.1, "3" stands for OD_{600} of 0.02, "4" stands for OD_{600} of 0.004, "5" stands for OD_{600} of 0.0008. B) Alcian Blue staining of the cells treated with robenidine. After robenidine treatment in RPMI medium at 37 ℃, cells were stained with Alcian Blue and photos were taken after centrifugation (left). The OD_{620} of supernatant was then measured by a plate reader. Left panel: Photos of cells stained with Alcian Blue; Right panel: percentage of the Alcian Blue dye binding to the cells. Error bars represent SDs of 6 technical replicates. ****p<0.0001. C) Quantitative RT-PCR analysis of *RLM1* expression after treatment of 8 µM robenidine for 90 minutes, 3 hours, 6 hours, 9 hours at 37 ℃. Error bars indicate standard deviation based on three technical replicates. Both experiments have been performed for at least 3 biological replicates.

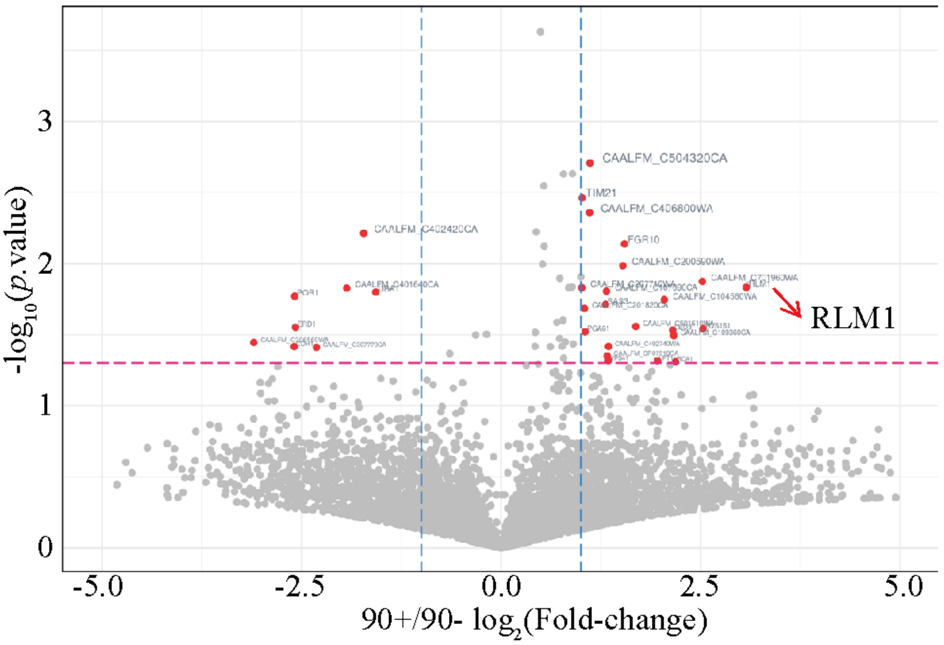

Figure S4. RNA-seq analysis of cells treated with robenidine for 90 minutes. Cells treated with robenidine for 90 minutes are extracted for RNA and send for sequencing. The red arrow indicates the target gene-*RLM1*. All the raw data are uploaded to NCBI.

References

1. Chen, C., and Noble, S.M. (2012). Post-transcriptional regulation of the Sef1 transcription factor controls the virulence of *Candida albicans* in its mammalian host. *PLoS pathogens* 8(11)**,** e1002956-e1002956. doi: 10.1371/journal.ppat.1002956.
2. Li, Y., Li, H., Sui, M., Li, M., Wang, J., Meng, Y., et al. (2019). Fungal acetylome comparative analysis identifies an essential role of acetylation in human fungal pathogen virulence. *Communications biology* 2**,** 154-154. doi: 10.1038/s42003-019-0419-1.
3. Romo, J.A., Zhang, H., Cai, H., Kadosh, D., Koehler, J.R., Saville, S.P., et al. (2019). Global transcriptomic analysis of the *Candida albicans* response to treatment with a novel inhibitor of filamentation. *mSphere* 4(5)**,** e00620-00619. doi: 10.1128/mSphere.00620-19.
4. Wang, S.-S., Zhou, B.O., and Zhou, J.-Q. (2011). Histone H3 lysine 4 hypermethylation prevents aberrant nucleosome remodeling at the *PHO5* promoter. *Molecular and cellular biology* 31(15)**,** 3171-3181. doi: 10.1128/MCB.05017-11.
5. Wang, X., Bing, J., Zheng, Q., Zhang, F., Liu, J., Yue, H., et al. (2018). The first isolate of *Candida auris* in China: clinical and biological aspects. *Emerging microbes & infections* 7(1)**,** 93-93. doi: 10.1038/s41426-018-0095-0.
6. White, T.C. (1997). Increased mRNA levels of *ERG16*, *CDR*, and *MDR1* correlate, with increases in azole resistance in *Candida albicans* isolates from a patient infected with human immunodeficiency virus. *Antimicrobial Agents and Chemotherapy* 41(7)**,** 1482-1487.
7. Zhai, P., Song, J., Gao, L., and Lu, L. A sphingolipid synthesis-related protein OrmA in *Aspergillus fumigatus* is responsible for azole susceptibility and virulence. *Cellular Microbiology* 0(0)**,** e13092. doi: 10.1111/cmi.13092.
